# Serum apolipoprotein B/apolipoprotein A1 ratio in relation to intervertebral disk herniation: a cross-sectional frequency-matched case–control study

**DOI:** 10.1186/s12944-021-01502-z

**Published:** 2021-07-29

**Authors:** Fei Chen, Tongde Wu, Chong Bai, Song Guo, Wenjun Huang, Yaqin Pan, Huiying Zhang, Desheng Wu, Qiang Fu, Qi Chen, Xinhua Li, Lijun Li

**Affiliations:** 1 Department of Cardiovascular, Pingxiang Hospital of Southern Medical University, Pingxiang, Jiangxi Province 337055 China; 2 Department of Spinal Surgery, Shanghai East Hospital, Tongji University School of Medicine, 150 JiMo Road, Shanghai, 200120 People’s Republic of China; 3 Department of Orthopedics, Shanghai General Hospital, Shanghai Jiao Tong University School of Medicine, Shanghai, 200080 People’s Republic of China; 4 Department of Cardiovascular, The Second Affiliated Hospital of Nanchang University, Nanchang, 330000 Jiangxi Province People’s Republic of China

**Keywords:** Apo B/Apo AI, Dyslipidemia, Intervertebral disk herniation, Lp(a), Serum lipid

## Abstract

**Study design:**

This was a cross-sectional frequency-matched case–control study.

**Background and aim:**

The serum lipid profile of lipoprotein(a) [Lp(a)] level and apolipoprotein B/apolipoprotein A1 ratio (Apo B/Apo A1) ratio were found to be more representative for serum lipid level and were recognized as the independent risk factors for various diseases. Although the blood levels of total cholesterol (TC), triglycerides (TG), low-density lipoprotein cholesterol (LDL-C), and high-density lipoprotein cholesterol (HDL-C) were found to be associated with symptomatic intervertebral disk herniation (IDH), no studies to date have evaluated the association of Apo AI, Apo B, Lp(a), and Apo B/Apo AI levels with symptomatic IDH. This study aimed to assess the link between blood lipid levels and symptomatic IDH.

**Method:**

The study included 1839 Chinese patients. Of these, 918 patients were diagnosed with IDH and enrolled in the experimental group. A control group of 921 patients underwent a physical examination during the same period. The serum lipid levels of TC, TG, LDL-C, HDL-C, Lp(a), Apo B, and Apo B/Apo AI were examined and analyzed. The control group comprised randomly selected patients who met the baseline levels of the aforementioned lipid molecules.

**Results:**

Patients with IDH exhibited significantly higher TC, TG, LDL, Apo B, and Lp(a) levels than controls. The percentage of high TC, high TG, high LDL, high Apo B, and high Lp(a) were obviously higher in the IDH group than in the control group. However, hyperlipidemia had no relationship with the degenerated segment of the IDH (*P* = 0.201). The odds ratio (OR) for the incidence of IDH with elevated levels of LDL-C, TC, TG, Lp(a), Apo B, and Apo B/Apo AI was 1.583, 1.74, 1.62, 1.58, 1.49, and 1.39, respectively. The correlation analysis revealed the correlation between elevated LDL-C, TC, TG, Apo B, Lp(a), and incidence of IDH was significant (*R*^2^_LDL_ = 0.017; *R*^2^_TC_ = 0.004; *R*^2^_TG_ = 0.015; *R*^2^_Apo B_ = 0.004; *R*^2^_Lp(a)_ = 0.021) (*P* < 0.05).

**Conclusion:**

This study suggested that elevated levels of serum TC, TG, LDL, Apo B, Lp(a), and Apo B/Apo AI were associated with a higher risk of IDH. This study provided useful information to identify a population that might be at risk of developing IDH based on elevated lipid levels.

## Introduction

Intervertebral disk herniation (IDH) is the main cause of disability, especially work-related disability [1–3]. Pathophysiological mechanisms underlying IDH remain unclear [3–8]. Many risk factors, including aging, injury, smoking, abnormal metabolism levels, and genetic risk, have been found to contribute to the initiation and development of IDH [9–11]. Among these factors, abnormal lipid metabolism and atherosclerosis (AS) have been implicated in the development of symptomatic IDH [12–16]. A Finnish study performed by Leino-Arjas [12] et al. identified a positive correlation between higher levels of total cholesterol (TC), low-density lipoprotein cholesterol (LDL-C), triglycerides (TG), and sciatica. Several studies further demonstrated that triglycerides (TG) and TC were related to the severity of low back pain and symptomatic IDH [13–18].

With the increased research on serum lipids, the lipoprotein fraction of apolipoprotein AI (Apo AI) and apolipoprotein B (Apo B) and the ratios of apolipoprotein B/apolipoprotein AI (Apo B/Apo AI), and lipoprotein(a) [Lp(a)] have received considerable attention in investigating dyslipidemia-related diseases in recent years; Apo B/Apo AI and Lp(a) were recognized as the independent risk factors for various diseases, including osteoarthritis and AS [19–21]. However, the possible association between Apo AI, Apo B, Lp(a), and symptomatic IDH remains undiscovered.

In this study, a frequency-matched case–control study of blood lipid levels of patients with symptomatic IDH was conducted to assess the link between blood lipid levels and symptomatic IDH.

## Methods

**Fig. 1:**
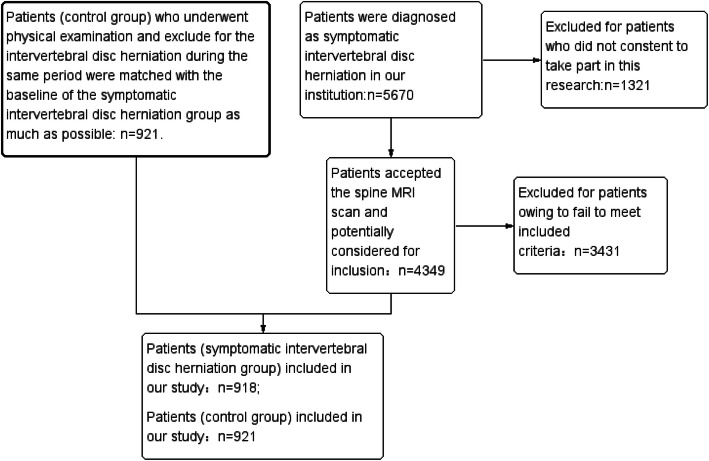
The flow-chart of including and excluding

All procedures in the present study were approved by our ethics committee. All patients signed the written informed consent. The detailed primary flow charts are presented in Fig. 1. A total of 4349 patients accepted magnetic resonance imaging (MRI) scanning and were potentially considered for inclusion from 2010 to 2019 in the institution. A total of 3431 patients were excluded because they did not meet the inclusion criteria: history of spinal disorders (*n* = 155), multiple IDHs (*n* = 367), spondylolysis (*n* = 98), foraminal or central canal stenosis (*n* = 187), spinal trauma (*n* = 67), primary osteoarthritis of the operated and/or contralateral joint (*n* = 367), inflammatory joint disease (*n* = 211), diabetes (*n* = 311), coronary heart disease (*n* = 516), cerebrovascular disease (*n* = 217), hypertension (*n* = 467), and smoking (*n* = 468). Further, 918 patients (399 men and 519 women; mean age: 60.74 ± 12.69 years, range 18–93 years) met the inclusion criteria and were included in the study in group 1 (symptomatic IDH group). A total of 921 patients (control group) (401 men and 520 women; mean age: 61.02 ± 12.59 years, range 18–91 years), who underwent a physical examination and an MRI scan during the same period, were matched with the baseline of the symptomatic IDH group (Table 1). The patients in the control group were excluded for intervertebral disk disease (IVDD) as detected by MRI [22, 23]. The procedure of selecting patients in these two groups and diagnosis were performed by experienced spine surgeons who did not know the purpose of the study. No statistically significant differences were found in age, sex, labor intensity, waist circumference, blood pressure, exercise habits, and body mass index (BMI) between the two groups (*P* > 0.05). This study has been reported according to the strengthening the reporting of cohort studies in surgery (STROCSS) criteria [24].

**Table 1:** Baseline characteristics of participants (*N* = 1839)

Variables	Group1(disc herniation) *n* = 918	Group2(control group) *n* = 921	*P* value
Gender(M/F)	(399/519)	(401/520)	0.889
Age (years)	60.74 ± 12.69	61.02 ± 12.59	0.967
Range (years)	18–93	18–91	
BMI (kg/m2)	22.40 ± 2.61	23.10 ± 2.72	0.637
WC [cm]	86.74 ± 1.73	87.72 ± 1.46	0.616
SBP (mm Hg)	123.30 ± 12.31	121.80 ± 13.42	0.504
DBP (mm Hg)	76.80 ± 8.56	77.32 ± 8.32	0.234
Exercise habits			
≥Once per week(%)	33.2	35.3	0.182

F: female M: male; WC: waist circumference; SBP: systolic blood pressure; DBP: diastolic blood pressure; A chi-square test and unpaired *t*-tests were used for analysis. *P < .05. NS = not statistically significant

### Patient selection

Patients in group 1 with (a) symptomatic cervical spondylotic myelopathy and thoracic and lumbar IDH were enlisted in the study. The diagnosis of cervical spondylotic myelopathy and thoracic and lumbar IDH was conducted on the basis of clinical presentation, physical examination, radiography, electromyography, computerized tomography, and/or MRI. (b) Also, patients with no symptom relief despite adequate medical treatment were included.

The inclusion criteria for group 2 were as follows: (1) The patients who underwent physical examination in the same period and were excluded for IVDD from 2009 to 2019 [2] (The patients in control group do not have any symptomatic of cervical spondylotic myelopathy, thoracic and lumbar IDH. Their will receive MRI examination once they have any symptoms of IDH). (2) Patients who were frequency-matched with group 1 by age and sex were included (Table 1).

The exclusion criteria for groups 1 and 2 were as follows: history of spinal disorders, multiple IDHs, spondylolysis, spondylolisthesis, foraminal or central canal stenosis, spinal trauma, spondyloarthritis, primary osteoarthritis of the operated and/or contralateral joint, inflammatory joint disease, diabetes, coronary heart disease, cerebrovascular disease, hypertension, smoking, and age less than 18 years [16].

### Definition of symptomatic IDH

#### Cervical disk herniation [25]

(1) Typical sensorial nerve root symptoms (pain or sense abnormity) were always present. (2) Motion (weakness and atrophy), sense (hypesthesia or paresthesia), and reflex (weakened or absent tendon reflex), if present, were confined to one dermatome and/or myotome that corresponded to pain and/or paresthesias; positive signs: a positive Spurling’s test, Eaten test, and Hoffman sign. (3) Clinical symptoms and physical signs were related to the degree of root compression caused by disk herniation and/or spondylotic lateral stenosis.

#### Thoracic disc herniation [26]

1. Localized axial back pain and/or axial back pain with radiation into the lumbar spine; sensory impairment; (2) special nerve root irritation signs: the pain can even simulate heart disease and/or shoulder pain and/or abdominal pain; (3) neurologic deficit: paraparesis and monoparesis; spasticity and hyperreflexia; bladder dysfunction (knee jerk or ankle reflex).

#### Lumbar disk herniation [16]

1. Low back pain with unilateral or bilateral lower limb radicular pain; (2) Bragard’s sign: straight leg raising test and strengthening test were positive; (3) Neurologic deficit: myasthenia, numbness, and/or disappearance of reflex (knee jerk or ankle reflex).

#### Imaging diagnosis

All the patients enrolled in the present study underwent the spine examination with a 1.5 T or 3.0 T MRI scanner. Both T2- and T1-weighted images were combined for evaluating the IVDD from C1/2 to L5/S1 regions by an experienced spine surgeon who knew nothing about the study. The degree of IVDD on MRI was based on the Pfirrmann grade system [25]. The criteria for grade 1 were as follows. Structurally, it was homogeneous and bright white in color. The distinction between the nucleus and the annulus was obvious. The disk signal strength was hyperintense and isointense compared with the cerebrospinal fluid. The intervertebral disk height was normal. The criteria for grade 2 were as follows: Its structure was nonhomogeneous in the absence and/or presence of horizontal bands. The distinction between the nucleus and the annulus was obvious. The disk signal strength was hyperintense and isointense compared with the cerebrospinal fluid. The intervertebral disk height was normal. The criteria for grade 3 were as follows. Structurally, it was nonhomogeneous and gray in color. The distinction between the nucleus and the annulus was not clear. The disk signal strength was medium. The intervertebral disk height was normal to marginally reduced. The criteria for grade 4 were as follows. Structurally, it was nonhomogeneous and gray to black in color. The distinction between the nucleus and the annulus disappeared. The signal intensity for the disk was intermediate to hypointense; The height of the intervertebral disk was normal to marginally reduced. The criteria for grade 5 were as follows. Structurally, it was nonhomogeneous and black in color. The distinction between the nucleus and the annulus disappeared. The signal strength for the disk was hypointense. The disk space collapsed [27–29].

#### Blood examination

All blood samples were collected in the same way between 07.30 and 08.30 a.m. after midnight fast. Biochemical analyses of blood samples were conducted on fresh specimens. Fasting serum samples were collected, and 5 mL of each sample was centrifuged at 4000 rpm for 6 min. The serum was collected from the samples, and the concentrations of TC, TG, LDL-C, high-density lipoprotein cholesterol (HDL-C), Apo AI, Apo B, and Lp(a) were measured in the same way with an automatic biochemical analyzer. The normal levels of the following indexes exhibited the following range: TC from 0 to 5.2 mmol/L; TG from 0 to 1.7 mmol/L; LDL-C from 0 to 3.4 mmol/L; HDL-C from 0.7 to 2.0 mmol/L; ApoEA1 from 1 to 1.6 g/L; Lp(a) from 0 to 30 mg/mL; and Apo B from 0.6 to 1.1 g/L. The normal value of the ratio of Apo B/Apo AI was 0.87 for the male participants and 0.65 for the female participants [30].

### Statistical analysis

**Fig. 2:**
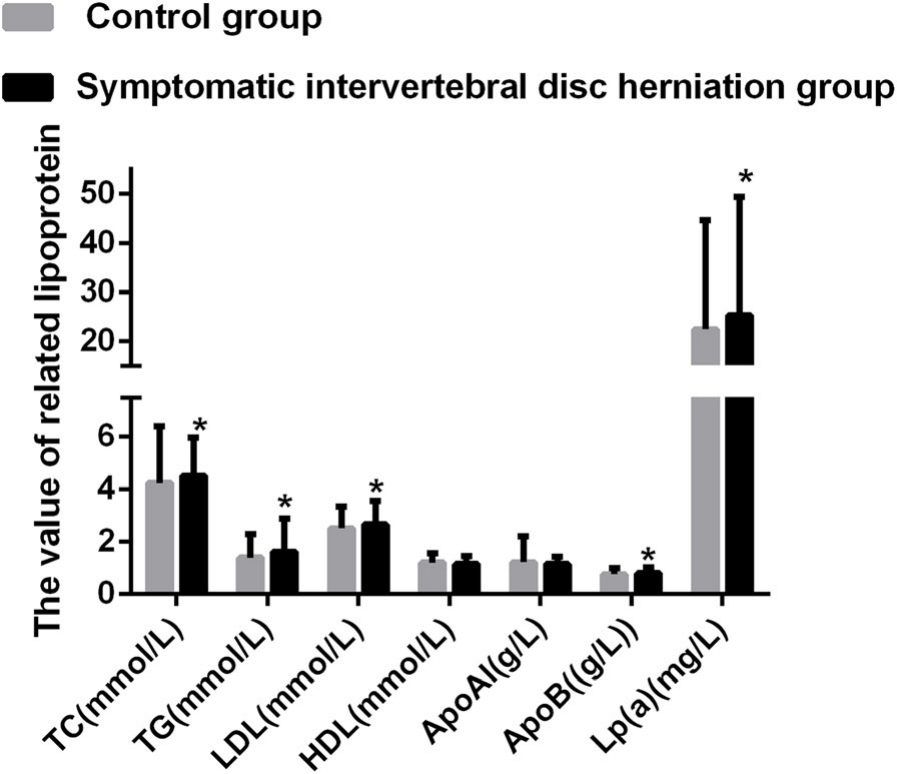
IDH patients exhibited significantly higher TC, TG, LDL, Apo B, Lp(a), and Apo B/Apo AI levels

Continuous variables were expressed as mean ± standard deviation and analyzed with unpaired-sample *t* tests. Categorical variables were expressed as percentages and analyzed with a chi-square test. The normality analysis was carried out for the continuous variables. SPSS (Version 20.0) was used for all statistical analyses. The data of continuous variables in the present study followed an ordinary normal distribution. The adjustment for multiple comparisons was conducted in the study. A *P* value lower than 0.007 (*P* < 0.007) indicated a statistically significant difference following the adjustment for multiple comparisons (Table 1 and Fig. 2). Multivariate logistic regression was used to assess the effects of blood lipids on symptomatic IDH. The effect indicators were odds ratio (OR) and 95% confidence interval. The correlation analysis was carried out in the present study. A *P* value lower than 0.05 indicated a statistically significant difference.

## Results

### IDH patients exhibited significantly higher levels of TC, TG, LDL, Apo B, Lp(a), and Apo B/Apo AI

The serum levels of TC, TG, LDL-C, HDL-C, Apo AI, Apo B, and Lp(a) were measured in all patients (Table 2 and Fig. 2). The patients with symptomatic IDH had significantly higher levels of TC (*P* = 0.002), TG (*P* = 0.00), LDL-C (*P* = 0.00), Apo B (*P* = 0.00), and Lp(a) (*P* = 0.006). No statistically significant differences were noted in HDL-C (*P* = 0.125) and Apo AI levels (*P* = 0.326). The ratios of Apo B/Apo AI were higher in patients with symptomatic IDH compared with the controls (0.78 ± 0.33 vs 0.71 ± 0.25, *P* < 0.01).

**Table 2:** The concentrations of serum lipids in two groups

Serum lipids	Disc herniation	Control group	*P* value
TC (mmol/L)	4.50 ± 1.48	4.23 ± 2.18	0.002
TG (mmol/L)	1.63 ± 1.26	1.4 ± 0.91	< 0.0001
LDL (mmol/L)	2.67 ± 0.9	2.52 ± 0.83	< 0.0001
HDL (mmol/L)	1.16 ± 0.29	1.21 ± 0.36	0.125
ApoAI(g/L)	1.15 ± 0.27	1.22 ± 1.00	0.326
ApoB(g/L)	0.80 ± 0.22	0.76 ± 0.23	< 0.0001
ApoB/ApoA1	0.78 ± 0.33	0.71 ± 0.25	< 0.0001
Lp(a) (mg/mL)	25.35 ± 24.01	22.45 ± 21.11	0.006

TC: total cholesterol, TG: triglycerides, LDL-C: low-density lipoprotein cholesterol, HDL-C: high-density lipoprotein cholesterol; Lp(a): lipoprotein(a); A unpaired *t*-tests were used in analysis. *P < .05. NS = not statistically significant

### Percentage of high TC, high TG, high LDL, high Apo B, and high Lp(a) obviously increased in the IDH group

The percentage of dyslipidemia incidence in the control and IDH group was further investigated in the study. The percentage of high TC, high TG, high LDL-C, low HDL-C, Apo AI, Apo B, and Lp(a) was 31.19, 13.85, 20.17, 7.52,14.9, 30.8, and 28.9%, respectively, in group 1 compared with 23.33, 15.34, 14.8, 5.98, 13.6, 51.14, and 21.76% in group 2, respectively (Table 3). The percentage of high TC, high TG and high LDL-C, Apo B, and Lp(a) were significantly higher in the IDH group compared with the control group (*P* = 0.000, *P* = 0.00, *P* = 0.02, *P* = 0.000, and *P* = 0.000, respectively). No statistically significant differences were noted in the levels of HDL-C (*P* = 0.189) and Apo AI (*P* = 0.412).

**Table 3:** Incidences of dyslipidaemia in two groups

Serum lipid	Disc degeneration N(%)	Control N(%)	*P* vaule
Total dyslipidaemia	20.06%	15.34%	0.008
High-TC (mmol/L)	31.19%	23.33%	< 0.0001
High-TG (mmol/L)	13.85%	23.64%	0.007
High-LDL (mmol/L)	20.17%	14.80%	0.02
Low-HDL (mmol/L)	7.52%	5.98%	0.189
Low-ApoAI(g/L)	14.90%	13.60%	0.412
High-LP(a)	28.90%	21.76%	< 0.0001
High-ApoB(g/L)	30.80%	51.14%	< 0.0001

TC: total cholesterol, TG: triglycerides, LDL-C: low-density lipoprotein cholesterol, HDL-C: high-density lipoprotein cholesterol; Lp (a): lipoprotein(a); A chi-square test were used in analysis. *P < .05. NS = not statistically significant

### Association between serum lipid abnormalities and degree of IVDD

**Fig. 3:**
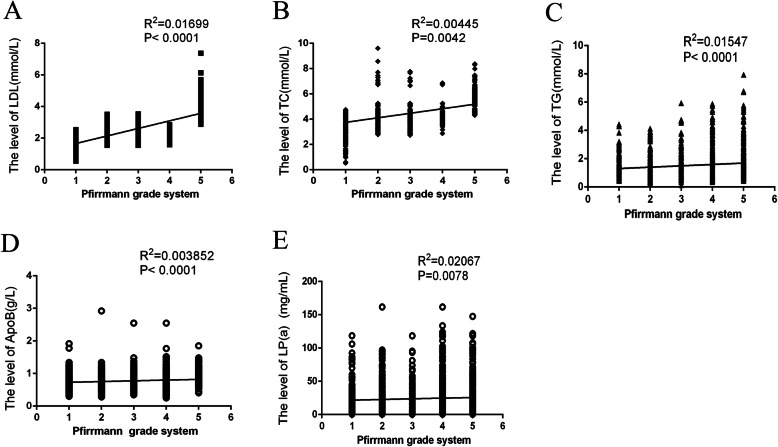
The association between serum lipid abnormalities and the grade of IVDD

The correlation analysis was conducted between serum lipid abnormalities and the degree of IVDD (Pfirrmann grade) to investigate further the correlation of symptomatic IDH with elevated levels of LDL-C, TC, TG, Lp(a), Apo B, and Apo B/Apo AI. As shown in Fig. 3, the correlation between elevated LDL-C, TC, TG, Apo B, Lp(a), and incidence of IDH were significant (*R*^2^_LDL_ = 0.017, *P* < 0.001; *R*^2^TC = 0.004, *P* < 0.004; *R*^2^_TG_ = 0.015, *P* < 0.001; *R*^2^_Apo B_ = 0.004, *P* < 0.001; *R*^2^_Lp(a)_ = 0.021, *P* < 0.008). These results showed that higher levels of LDL-C, TC, TG, Lp(a), Apo B, and Apo B/Apo AI were closely related to disk herniation.

### Hyperlipidemia did not influence the degenerated segment of the intervertebral disk

**Fig. 4:**
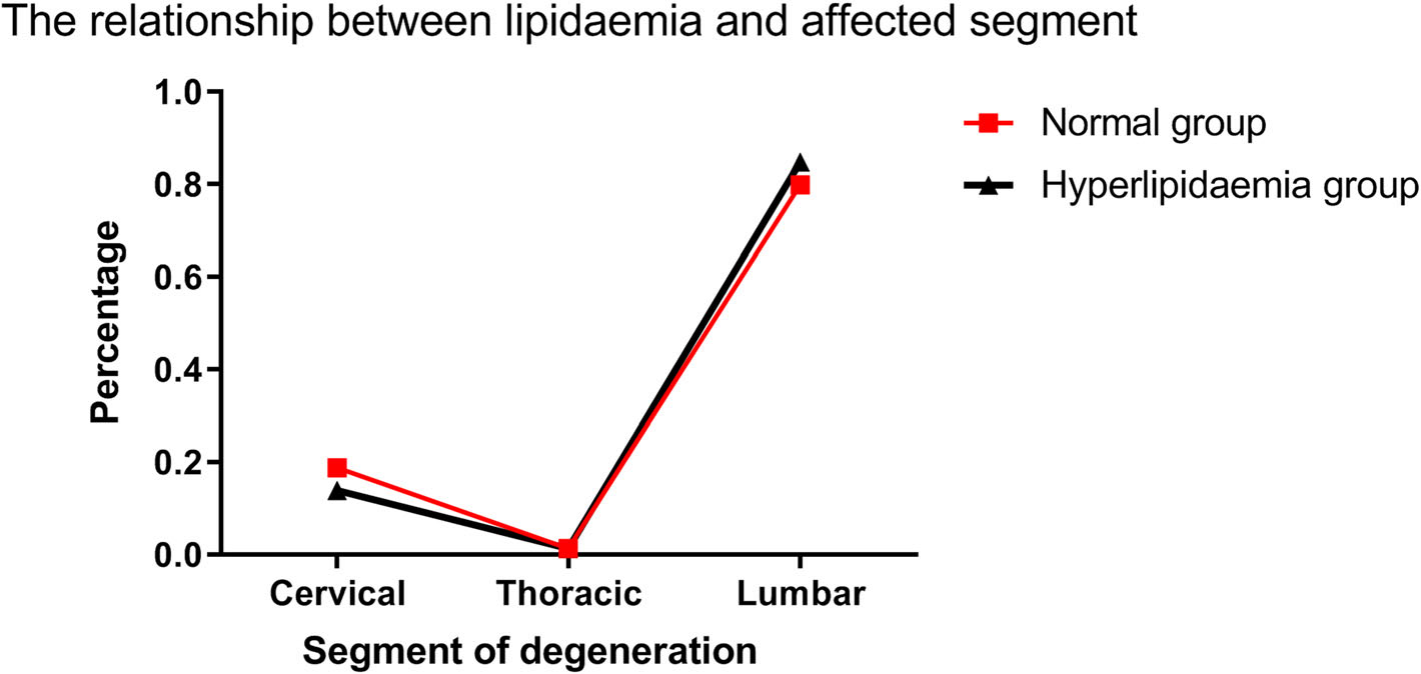
Hyperlipidaemia did not affect the segment of degenerated intervertebral disc

The categorical data of the patients with disk herniation were analyzed to explore the relationship between hyperlipidemia and the disk segment in the intervertebral disk group. The hyperlipidemia group (*n* = 689) exhibited the following percentages of degenerated segments in the cervical, thoracic, and lumbar regions: 13.9, 1.3, and 84.8%, respectively. Compared with the hyperlipidemic samples, the participants with normal serum lipid levels exhibited the incidence of 18.8, 1.3, and 79.9% that corresponded to the cervical, thoracic, and lumbar regions, respectively (*n* = 229). No significant differences in the herniation segments were noted between these two groups (*P* = 0.201) (Fig. 4).

The categorical data were further analyzed to identify the relationship between the blood lipid levels and the segment of disk herniation in the cervical and lumbar regions. Considering the small sample of the affected segment in the thoracic disk herniation, the study analyzed only the serum lipid levels and segment of disk herniation in the cervical and lumbar regions.

**Fig. 5:**
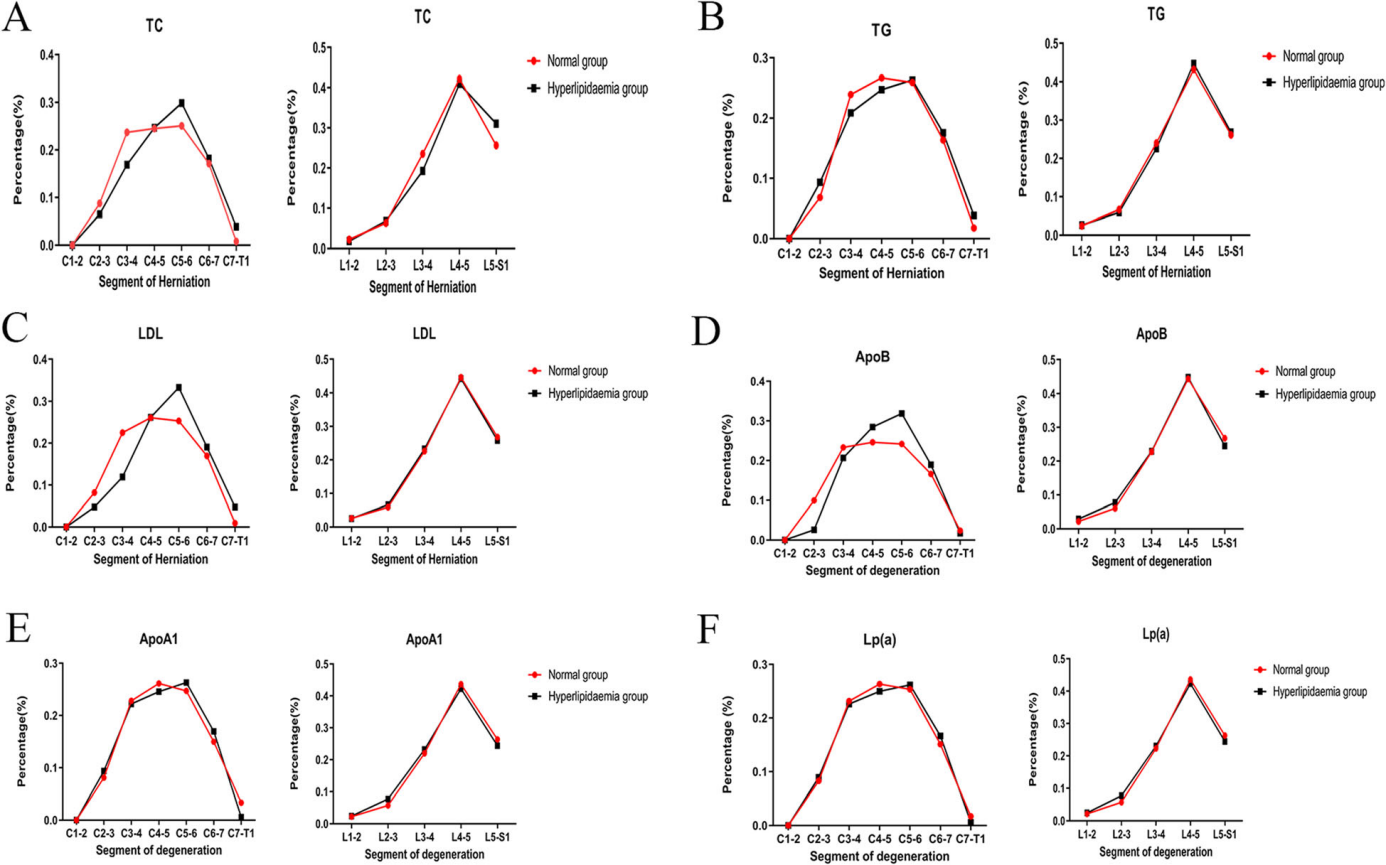
Hyperlipidaemia did not affect the incidence of intervertebral disc degenerated segment in cervical and lumbar spine

The value of the total segments in the cervical, thoracic, and lumbar regions was 137, 12, and 769, respectively. No significant differences were noted between serum lipid levels in the C3–C4 (*P* = 0.282) and C5–C6 (*P* = 0.373) segments with regard to TC levels (Fig. 5A). Similarly, no significant differences were observed in the C3–C4 (*P* = 0.108) and C5–C6 segments with regard to LDL-C levels (*P* = 0.254) (Fig. 5C). With regard to the levels of Apo B, the C5–C6 segment in the hyperlipidemia group (31.9%) was higher than that in the normal group (24.2%), although no significant differences were noted (Fig. 5D, *P* = 0.2). With regard to Apo A1 (Fig. 5E), Lp(a) (Fig. 5F) and triglycerides (TG) (Fig. 5B), the distribution of herniation segments in the hyperlipidemia and control groups exhibited similar trends in both the lumbar and cervical segments. However, the study achieved no statistical significance. Compared with cervical and lumbar IDH, the incidence of thoracic IDH was quite low. The relatively small sample size in the study might contribute to the no-significance result.

### Patients with elevated levels of LDL-C, TC, TG, Lp(a), Apo B, and Apo B/Apo AI exhibited a higher risk of disk herniation

A multivariate logistic regression analysis was performed to identify further the risk for the incidence of a symptomatic IDH with elevated LDL-C, TC, TG, Lp(a), Apo B, and Apo B/Apo AI levels. As shown in Table 4, the results showed that higher LDL-C, TC, TG, Lp(a), Apo B, and Apo B/Apo AI levels exhibited a higher risk of disk herniation.

**Table 4:** Multivariate logistic regression of multiple covariates and the risk of intervertebral disc degeneration

Variables	Risk of intervertebral disc degeneration		
	OR	95% CI	*P* value
LDL	1.58	1.427–1.796	0.001
Triglycerides (TG)	1.62	1.295–2.023	< 0.0001
Total cholesterol (TC)	1.74	1.282–2.365	< 0.0001
ApoB	1.49	1.346–1.661	0.001
ApoB/ApoAI	1.39	1.254–1.595	< 0.0001
LP(a)	1.58	1.255–1.975	< 0.0001

TC: total cholesterol, TG: triglycerides, LDL-C: low-density lipoprotein cholesterol, HDL-C: high-density lipoprotein cholesterol; LP(a): lipoprotein(a); OR odds ratio, CI confidence interval; The multivariate logistic regression was used to this analysis. *P < .05. NS = not statistically significant

## Discussion

The relationship between blood lipid and IVDD-related disease has been examined by a multitude of studies [12–16]. Higher levels of TC, LDL-C, and TG have been shown to be associated with sciatica [12], back pain, and/or disk herniation [13–16]. In agreement with these results, the data showed that patients with IDH exhibited significantly higher TC, TG, and LDL levels, and the percentage of high TC, high TG, and high LDL obviously increased in the IDH group.

ApoA1 is the main protein component of HDL particles, which plays a key role in reversing cholesterol transport and regulating inflammatory response and antioxidant processes [31]. Apo B is the main component of low-density lipoprotein (LDL) and very low-density lipoprotein (VLDL), representing atherogenic particles [32]. A previous study reported that an increased Apo B/Apo A1 ratio was a potential risk factor for osteonecrosis [33]. A number of clinical prospective studies have shown that the Apo B/ApoA1 ratio is the risk factor for cardiovascular, osteoarthritis, rheumatoid arthritis, and metabolic syndrome disease [19–21, 27, 34–36]. However, whether the levels of Apo AI, Apo B, Apo B/Apo AI, and Lp(a) are associated with symptomatic IDH is still unclear.

In the present study, the relationship between the levels of Apo AI, Apo B, and Lp(a), and symptomatic IDH was examined for the first time. As a result, the levels of Apo B and Lp(a) were shown to be positively associated with the incidence of symptomatic IDH. For the LDL-C, Apo B can facilitate cholesterol delivery to the tissues. However, Apo AI can facilitate the peripheral cell uptake of cholesterol and help in the transport of cholesterol to the liver for digestion. Thus, the levels of these Apo B/Apo AI can mirror cholesterol transport ability to the peripheral tissues and determine the level of cholesterol in plasma [33, 37]. Higher plasma levels of Apo B and an increased Apo B/Apo AI ratio in patients with symptomatic IDH suggested a prominent cholesterol transport to the peripheral tissues, including IVD, in these patients. Lp(a) shares the similar protein and lipid structure with LDL-C. Various studies have shown that high levels of Lp(a) in plasma can be a risk factor for cardiovascular disease, osteoarthritis, and RA [38]. The present study showed that the level of Lp(a) increased in the IDH group. This study was the first to report this finding in the field of symptomatic IDH.

The exact pathophysiologic mechanism underlying the relationship between blood lipid levels and lumbar disk herniation is still uncertain. The increased levels of TC, TG, LDL-C, Apo B, and Lp(a) in patients with IDH might be due to several reasons. First, the IVD is a barely vascularized region; its nutrient supply is through the blood capillaries of endplate cartilage and annulus fibrosis [29]. High levels of serum cholesterol [28], triglycerides [28, 39–41], LDL-C [42], Apo B, and Lp(a) contribute to AS. The presence of atherosclerotic plaques inhibits the vascular supply to the poorly vascularized IVD and induces IVDD/IDH [43]. In agreement with the hypothesis, the association between symptomatic IDH and AS was identified by a lot of studies. A study that included 86 people suggested that abdominal aorta AS and stenosis of segmental artery ostium played a vital role in symptomatic IDH [44]. Another 25-year follow-up study showed that the calcific atherosclerotic deposits in the abdominal aorta increased the risk of disk herniation and back pain [45]. Second, the activated inflammatory cells induced by high serum lipid levels might comprise an important pathway in developing symptomatic IDH for patients with hyperlipidemia. The activation of cytokines plays a significant role in the progression of IVDD/IDH [43, 46]. Pro-inflammatory cytokines were closely associated with blood lipid levels [5, 47]. Therefore, the elevated blood lipid levels might strengthen the inflammatory response and/or the level of systemic inflammation, in turn contributing to disk herniation. Third, the oxidized low-density lipoprotein (oxLDL) and the increased expression of lectin-like low-density lipoprotein receptor 1 (LOX-1) caused by dyslipidemia might also be related to the development of symptomatic IDH. A previous study suggested that [48] the levels of the oxLDL and LOX-1 positively correlated with the extent of IDH. The mechanism of action involved the increase in the LOX-1-induced expression of MMP3. This in turn caused the oxLDL to significantly reduce the viability of human nucleus pulpous. The production of oxLDL usually originates from LDL-C oxidized under oxidative stress conditions. Therefore, the elevated LDL-C levels can increase the level of oxLDL/LOX-1 and accelerate IVDD. However, whether serum oxLDL/LOX-1 are elevated in IVDD/IDH patient are still unclear. Further study to investigate the relationship between serum oxLDL/LOX-1 and IVDD/IDH are needed in the future.

Increased levels of TC, TG, LDL-C, Apo B, and Lp(a) and decreased level of HDL-C are atherogenic lipid markers. The management of the cardiovascular disease has traditionally focused on reducing serum lipid levels [49]. This study found that elevated serum lipid levels significantly correlated with IDH and high serum lipid levels foreshadowed a higher incidence of IDH. This association opened up a new way to reduce the risk of IDH/IVDD disease by controlling blood lipid levels. However, this study had several limitations. First, no data were provided regarding the levels of VLDL. Second, this was a retrospective case–control study, and therefore the causal relationships between serum lipid components and symptomatic IDH still remain unclear. Large, longitudinal follow-up observational and interventional studies are needed to prove the cause-and-effect relation and discover useful treatments for IVDD/IDH [16].

## Conclusions

The level of TC, TG, LDL-C, Apo B (Apo B/Apo AI), and Lp(a) positively correlated with the incidence of symptomatic IDH. However, hyperlipidemia had no effect on the degenerated segment of the intervertebral disk. This association provided novel evidence regarding the risk of symptomatic IDH disease are related with the elevated serum lipid levels and also indicated that a decrease in serum lipid levels could be a promising target for treating IDH. This study provided useful information to identify a population that might be at risk of developing disk herniation based on elevated lipid levels and helped to identify the possible mechanism of IVDD.

## Data Availability

The datasets used and/or analyzed during the current study are available from the corresponding author on reasonable request.
